# New Books

**Published:** 2011-01

**Acute Exposure Guideline Levels for Selected Airborne Chemicals, Vol 9**

Committee on Acute Exposure Guideline Levels; Committee on Toxicology; National Research Council

Washington, DC: National Academies Press, 2010. 462 pp. ISBN: 978-0-309-15944-9, $89.25

**African Climate and Climate Change**

Charles J.R. Williams, Dominic R. Kniveton, eds.

New York: Springer, 2011. 250 pp. ISBN: 978-90-481-3841-8, $129

**Arts: A Science Matter**

Maria Burguete, Lui Lam

Hackensack, NJ: World Scientific, 2011. 400 pp. ISBN: 978-981-4324-93-9, $78

**Bringing Ecologists and Economists Together**

T. Söderqvist, A. Sundbaum, C. Folke, K.-G. Mäler, K.-G., eds.

New York: Springer, 2011. 281 pp. ISBN: 978-90-481-9475-9, $129

**Ecology Revisited: Reflecting on Concepts, Advancing Science**

Astrid Schwarz, Kurt Jax, eds.

New York: Springer, 2011. 412 pp. ISBN: 978-90-481-9743-9, $209

**Expect the Unexpected: A First Course in Biostatistics**

Raluca Balan, Gilles Lamothe

Hackensack, NJ: World Scientific, 2011. 200 pp. ISBN: 978-981-4291-32-3, $54

**Ingested Nitrate and Nitrite, and Cyanobacterial Peptide Toxins**

IARC Monographs Vol. 94

Geneva: WHO Press, 2010. 457 pp. ISBN: 978-9-2832-1294-2, $55

**Order and Disorder: Science Essentials for the Non-Scientist**

Myron Kaufman

Hackensack, NJ: World Scientific, 2010. 400 pp. ISBN: 978-1-84816-574-8, $88

**Pathways for Getting to Better Water Quality: The Citizen Effect**

Lois Wright Morton, Susan S. Brown, eds.

New York: Springer, 2011. 273 pp. ISBN: 978-1-4419-7281-1, $129

**Persistent Pollution—Past, Present and Future**

Markus Quante, Ralf Ebinghaus, Götz Flöser, eds.

New York: Springer, 2011. 499 pp. ISBN: 978-3-642-17418-6, $179

**Perspectives from United Kingdom and United States Policy Makers on Obesity Prevention**

Paula Tarnapol Whitacre and Annina Catherine Burns, eds.

Washington, DC: National Academies Press, 2010. 96 pp. ISBN: 978-0-309-15078-1, $21

**Protecting Children’s Health in a Changing Environment**

WHO Regional Office for Europe

Geneva: WHO Press, 2010. 90 pp. ISBN: 978-9-2890-1419-9, $20

**The End of Energy: The Unmaking of America’s Environment, Security, and Independence**

Michael Graetz

Cambridge, MA: MIT Press, 2011. 384 pp. ISBN: 978-0-262-01567-7, $29.95

**The Environmental Politics of Sacrifice**

Michael F. Maniates, John M. Meyer

Cambridge, MA: MIT Press, 2010. 344 pp. ISBN: 978-0-262-51436-1, $25

**The Fate of Greenland: Lessons from Abrupt Climate Change**

Philip W. Conkling

Cambridge, MA: MIT Press, 2011. 224 pp. ISBN: 978-0-262-01564-6, $29.95

**The Human Genome, 3rd ed., A User’s Guide**

Julia E. Richards, R. Scott S.H. Hawley

St. Louis, MO: Elsevier, 2011. 420 pp. ISBN: 978-0-12-333445-9, $89.95

